# OLDER ADULT HEALTH IN THE CITY OF CHICAGO

**DOI:** 10.1093/geroni/igz038.925

**Published:** 2019-11-08

**Authors:** Brittney S Lange Maia, Emily Laflamme, Fernando DeMaio, Raj C Shah

**Affiliations:** 1 Rush University Medical Center, Chicago, Illinois, United States; 2 Chicago Department of Public Health, Chicago, Illinois, United States; 3 DePaul University, Chicago, Illinois, United States; 4 Rush University, Chicago, Illinois, United States

## Abstract

In 2012, Chicago was designated as an Age Friendly City. However, city-wide data on the health and health disparities experienced by older adults have been scarce. In order to address this knowledge gap, the Chicago Department of Public Health (CDPH) partnered with the Center for Community Health Equity at Rush and DePaul Universities to create a report describing health status among adults age 65+. Data were from the Healthy Chicago Survey—a population-based health survey conducted by CDPH, the American Community Survey, Hospital Discharge Data, and State Vital Records. The report highlights considerable racial/ethnic diversity in Chicago, as 38% of older adults are white, 37% black, 18% Latinx, and 7% are Asian. Encouraging results exist regarding healthcare access; 96% have a personal health care provider and 89% report being able to get care needed through their health plan. Several areas of improvement are needed regarding root causes of health. More older adults live below the federal poverty level (15.9%) compared to the overall U.S (9.3%), and 45.8% would be unable to pay for an unexpected $400 expense. Disparities were evident as life expectancy at age 65 is 2.5 years longer for Latinx and white older adults (age 85) compared to African Americans (age 82.4). African American and Latinx older adults had higher rates of preventable hospitalizations per 10,000 (801.1 and 678.9, respectively) compared to white (492.4) and Asian (374.1) older adults. Findings from this report will spur Chicago’s continued progress as an Age-Friendly City for all its residents.

